# Donor-Derived Cell-Free DNA as a Non-Invasive Readout of Activity Across the Rejection Continuum

**DOI:** 10.3389/ti.2026.16099

**Published:** 2026-02-24

**Authors:** Louise Benning, Aylin Akifova, Bilgin Osmanodja, Christian Morath, Julia Beck, Ekkehard Schütz, Klemens Budde

**Affiliations:** 1 Department of Nephrology, Heidelberg University Hospital, Heidelberg, Germany; 2 Department of Nephrology and Intensive Care, Charité Universitätsmedizin Berlin, Berlin, Germany; 3 Department of Nephrology and Hypertension, Klinikum Nuremberg, Paracelsus Medical University, Nuremberg, Germany; 4 Chronix Biomedical GmbH, Göttingen, Germany; 5 Insight Molecular Diagnostics Inc., Nashville, TN, United States

**Keywords:** dd-cfDNA, donor-derived cell-free DNA, kidney transplantation, rejection, rejection continuum

## Abstract

Recent work demonstrated that kidney allograft rejection unfolds along a biological continuum that can be quantified using histopathology-derived continuous indices. To investigate whether donor-derived cell-free DNA (dd-cfDNA) reflects this rejection continuum and complements these histopathology-derived indices, we analyzed the association between dd-cfDNA measures and the newly developed rejection indices in 249 indication biopsies from two independent cohorts. dd-cfDNA was analyzed as percentage, absolute copies/mL, and as a previously developed combined continuous model (CM) score integrating both measures to mitigate limitations of relative measurements. dd-cfDNA increased with histopathological activity and was highest in biopsies with microvascular inflammation (MVI), including antibody-mediated (AMR) and mixed rejection, paralleling high AMR/MVI and activity indices. T-cell-mediated rejection (TCMR) showed elevated TCMR/tubulointerstitial inflammation (TI) indices but lower and more variable dd-cfDNA, accompanied by increased total cfDNA, providing a plausible explanation for the reduced detectability of low-grade TCMR when dd-cfDNA is expressed as a percentage alone. Interclass correlation analyses revealed the strongest associations between dd-cfDNA and the AMR/MVI and activity indices. The combined CM score achieved the highest overall associations (sum R^2^ = 3.4), outperforming absolute and relative dd-cfDNA measures. Thus, dd-cfDNA may serve as a non-invasive readout of graft inflammation and extends the rejection-continuum concept into the non-invasive space.

## Graphical Abstract

**Figure:**
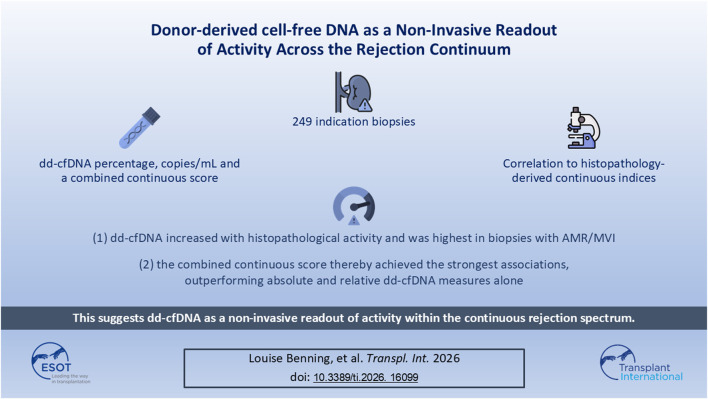

## Introduction

The recently published study by Vaulet et al. compellingly demonstrates that kidney allograft rejection unfolds along a biological continuum rather than within rigid diagnostic subcategories [1]. By deriving continuous activity and chronicity indices from assessed histological lesion scores in a cohort study of 19,500 biopsies from 8,873 patients across 10 centers worldwide, the authors elegantly quantified the spectrum of rejection and distinguished microvascular inflammation from tubulointerstitial inflammation patterns. This is a huge step forward in the interpretation of Banff lesions in a more quantitative manner and shall provide a better and standardized patient care if implemented as histopathological standard.

An important next step is determining whether non-invasive biomarkers map onto this continuum. Donor-derived cell-free DNA (dd-cfDNA) as an indicator of graft damage is now widely used in clinical kidney transplant care, in both for-cause and surveillance settings [2–4]. There is ongoing discussion about whether such a “liquid biopsy” may complement the histopathological and clinical assessment [3–7]. Usually, dd-cfDNA is measured as percentage of total cfDNA, which, however, may cause misinterpretation in patients with high or low total cfDNA [8]. Absolute dd-cfDNA copy numbers may address some of these limitations but require standardized measurement approaches [8, 9]. To address this, we recently developed a combined continuous model (CM) score that integrates relative and absolute dd-cfDNA measurements into a single composite metric, with the aim of reducing false classifications and improving the robustness of dd-cfDNA interpretation [10].

The aim of this study was therefore to determine whether, and how, dd-cfDNA measures including the combined CM score correlate with the newly developed histopathology-based continuous rejection indices [1] and to identify which Banff lesion patterns are the principal drivers of dd-cfDNA release.

## Methods

To this end, we analyzed a cohort of 249 indication biopsies from two previously published independent cohorts [11, 12]. Detailed descriptions of the patient populations have been reported previously. For each biopsy, histopathological diagnoses were assigned according to Banff criteria [13], and continuous rejection indices for antibody-mediated rejection/microvascular inflammation (AMR/MVI), T-cell-mediated rejection/tubulointerstitial inflammation (TCMR/TI), global activity, and chronicity were calculated as described by Vaulet et al. [1].

dd-cfDNA was analyzed as relative percentage of total cfDNA, as absolute copies per milliliter (cp/mL), and using the previously developed combined CM score integrating relative and absolute dd-cfDNA measurements [10]. Descriptive analyses were performed by histopathological diagnostic category, with indices and dd-cfDNA measures summarized as means ± standard deviations.

To assess associations between individual Banff lesion scores and cfDNA measures, Spearman rank correlation coefficients (ρ) were calculated between each lesion score and dd-cfDNA metrics (percentage, cp/mL, CM score) as well as total cfDNA. Lesions were grouped according to AMR-related, TCMR-related, and chronic injury categories [13].

To evaluate how well dd-cfDNA quantitatively tracks the continuous rejection indices across their dynamic range, interclass correlation analyses were performed. For each dd-cfDNA metric, biopsies were stratified into five equal-sized quintiles. Mean values and standard errors of dd-cfDNA measures and histopathology-derived indices were calculated within each quintile, and linear regression between quintile means was used to derive coefficients of determination (R^2^) and corresponding *P*-values. Overall explanatory association was compared across dd-cfDNA metrics by summing R^2^ values across indices.

Because the endpoints were continuous indices and ordinal lesion grades, Spearman’s ρ and R^2^ were used as descriptive measures of association. Our analyses were not intended to assess diagnostic discrimination or calibration for binary outcomes.

## Results

Across all dd-cfDNA measures, the highest dd-cfDNA levels were observed in biopsies with high histopathological activity indices (Table 1). Biopsies characterized by MVI, including AMR, mixed rejection, and DSA-negative/C4d-negative MVI, showed the highest AMR/MVI and global activity index values and correspondingly elevated dd-cfDNA levels. Biopsies with TCMR displayed elevated TCMR/TI and activity indices but lower and more variable dd-cfDNA levels. Borderline changes were associated with moderately increased TCMR/TI and activity indices, falling between overt TCMR and non-rejection. Categories characterized by chronic or non-immune-mediated injury, such as interstitial fibrosis/tubular atrophy (IFTA), glomerulonephritis (GN), or calcineurin inhibitor (CNI) toxicity, exhibited low dd-cfDNA and low activity despite variable chronicity indices.

**TABLE 1:** Indices and dd-cfDNA by histopathology.

Histopathological Diagnosis	N	AMR/MVI [index]	TCMR/TI [index]	Activity [index]	Chronicity [index]	dd-cfDNA [%]	dd-cfDNA [cp/mL]	dd-cfDNA [CM-Score]
AMR	46	5.04 ± 2.28	0.95 ± 0.85	4.85 ± 2	7.07 ± 3.52	2.01 ± 1.48	96.4 ± 99.8	0.46 ± 0.63
TCMR	16	0.43 ± 0.79	3.57 ± 1.25	4.56 ± 2.1	3.25 ± 2.65	0.56 ± 0.39	53.4 ± 84.4	−0.14 ± 0.52
Mixed Rej	8	5.29 ± 0.96	4.47 ± 1.27	9.13 ± 1.64	6.13 ± 3	1.82 ± 1.11	153.6 ± 111.6	0.73 ± 0.47
DSA- MVI	9	5.34 ± 2.17	1.31 ± 1.04	5.78 ± 2.22	6.22 ± 3.9	1.81 ± 1.41	113.2 ± 101.9	0.5 ± 0.63
Borderline	20	0.6 ± 0.86	1.87 ± 0.23	2.7 ± 0.73	2.85 ± 2.54	0.65 ± 0.54	46.1 ± 66.1	−0.16 ± 0.51
IFTA	46	0.34 ± 0.59	0.64 ± 0.63	0.8 ± 1.09	3.61 ± 1.95	0.41 ± 0.52	21.4 ± 28.4	−0.4 ± 0.33
GN	20	0.57 ± 1.18	1.23 ± 1.2	1.5 ± 1.57	5.1 ± 2.83	0.33 ± 0.2	14.5 ± 9.6	−0.46 ± 0.15
CNI Tox	20	0.47 ± 0.9	0.33 ± 0.37	0.35 ± 0.67	3.85 ± 2.46	0.58 ± 0.59	14.8 ± 11.2	−0.38 ± 0.27
UTI	6	1.16 ± 1.34	2.66 ± 1.85	3.67 ± 3.27	5.83 ± 3.54	0.51 ± 0.32	14.2 ± 6.3	−0.38 ± 0.16
BKVAN	19	0.89 ± 1.2	2.72 ± 1.6	4.11 ± 2.54	2.84 ± 2.06	0.56 ± 1.04	56.4 ± 88.7	−0.2 ± 0.68
Normal	14	0.16 ± 0.58	0.26 ± 0.47	0.21 ± 0.43	1.29 ± 2.37	0.6 ± 1.02	29.8 ± 47.7	−0.31 ± 0.5
ATI	8	0.1 ± 0.27	0.36 ± 0.38	0.38 ± 0.52	1.25 ± 1.04	0.87 ± 1.04	92.3 ± 101.7	0.12 ± 0.77
Other	17	0.45 ± 1.0	1.07 ± 1.61	1.35 ± 1.84	2.71 ± 2.47	0.32 ± 0.11	37.1 ± 49.5	−0.29 ± 0.31

Averages and standard deviations are given. AMR, antibody-mediated rejection; ATI, acute tubular injury; BKVAN, BK polyomavirus nephropathy; CM-score, combined model score; CNI Tox, calcineurin inhibitor toxicity; dd-cfDNA, donor-derived cell-free DNA; DSA, donor-specific antibodies; GN, glomerulonephritis; IFTA, interstitial fibrosis and tubular atrophy; MVI, microvascular inflammation; Rej, rejection; TCMR, T-cell-mediated rejection; TI, tubulointerstitial inflammation; UTI, urinary tract infection.

**FIGURE 1:**
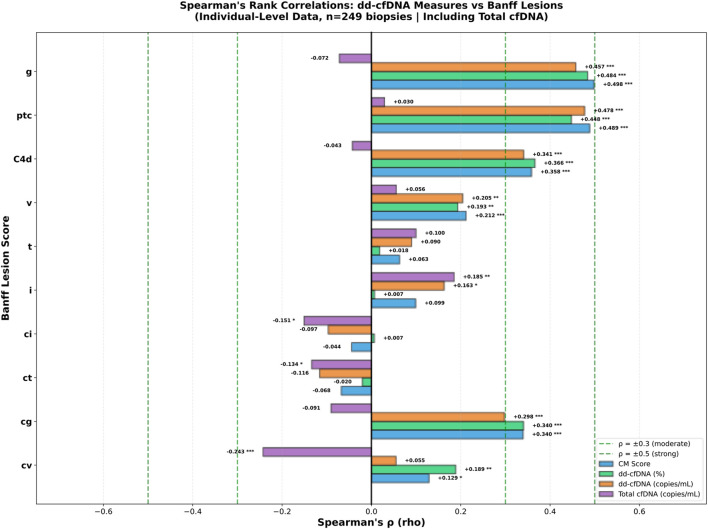
Spearman rank correlations between individual Banff lesion scores and circulating cfDNA measures in 249 kidney transplant biopsies. Correlations are shown for donor-derived cell-free DNA (dd-cfDNA) expressed as combined continuous model (CM) score, percentage, and copies/mL, as well as total cfDNA. dd-cfDNA shows strongest associations with microvascular inflammation lesions (g, ptc), whereas correlations with tubulointerstitial and chronic lesions are weaker. Dashed lines indicate thresholds for moderate and strong correlations; statistical significance is indicated as *P* < 0.05 (*), *P* < 0.01 (**), and *P* < 0.001 (***).

To further assess the contribution of individual Banff lesions to cfDNA release, Spearman correlation coefficients were calculated between each lesion score and dd-cfDNA measured as percentage, copies/mL, and combined CM score, as well as total cfDNA (Figure 1). Lesions associated with AMR showed the highest correlation coefficients with dd-cfDNA measures, with glomerulitis (g) and peritubular capillaritis (ptc) demonstrating the largest coefficients. For TCMR lesions, correlations with dd-cfDNA percentage were lower, whereas higher coefficients were observed with absolute dd-cfDNA copies/mL and total cfDNA. Chronic lesions showed lower correlation coefficients overall with dd-cfDNA: among chronic Banff lesions, only transplant glomerulopathy (cg) and, to a lesser extent, vascular fibrous intimal thickening (cv) were associated with increased dd-cfDNA levels, whereas interstitial fibrosis (ci) and tubular atrophy (ct) showed no positive association or an inverse relationship.

**FIGURE 2:**
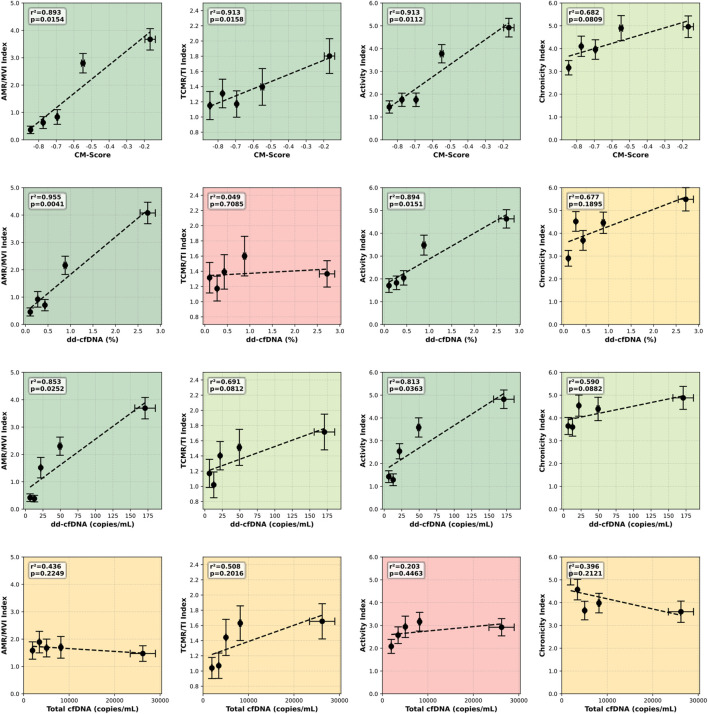
Interclass correlations between continuous indices of kidney transplant rejection and circulating cfDNA measures in 249 kidney transplant biopsies. Biopsies were stratified into quintiles based on the four cell-free DNA (cfDNA) measures, including the combined continuous model (CM) score, donor-derived cell-free DNA (dd-cfDNA) percentage, dd-cfDNA copies/mL, and total cfDNA (rows, top to bottom). Mean values of (dd-)cfDNA and histopathology-derived indices (AMR/MVI, TCMR/TI, activity, chronicity) are shown across quintiles, with linear regression used to derive R^2^ and P-values. The CM score showed the highest overall explanatory power compared with absolute and relative (dd-)cfDNA measures. Background colors indicate statistical significance.

Building on this descriptive alignment, we next assessed how well dd-cfDNA quantitatively tracks the newly developed indices across their dynamic range. We therefore performed interclass (IC) correlations between the histopathology indices and the dd-cfDNA measures, with classes defined by quintiles (20% intervals) of the dd-cfDNA levels in the cohort. Across all dd-cfDNA measurements, the highest R^2^ in the IC correlations were observed for the AMR/MVI and activity indices (Figure 2). In contrast, R^2^ values for the TCMR/TI and chronicity indices were lower (Figure 2). Among the dd-cfDNA metrics, the combination score, which simultaneously considers percent and absolute dd-cfDNA, showed the highest overall association with the indices, with a sum R^2^ of 3.4 across all indices, followed by absolute dd-cfDNA levels in cp/mL (2.9) and relative dd-cfDNA percentages (2.6) (Figure 2). Supplementary Figure S1 shows the individual-biopsy relationships between each cfDNA measure and the histopathology-derived continuous rejection indices.

## Discussion

In this study, we evaluate the newly developed histopathology-derived continuous indices [1] in an independent cohort of clinically indicated biopsies and demonstrate how dd-cfDNA aligns with the inflammatory component of the rejection continuum. Together, our findings suggest that dd-cfDNA may serve as a non-invasive readout of the acuity of graft injury within the continuous rejection spectrum.

As expected, biopsies with MVI as seen in AMR, mixed rejection, and DSAnegC4dneg MVI showed the highest AMR/MVI and activity index values, along with the highest dd-cfDNA levels, highlighting once again the utility of dd-cfDNA, particularly in AMR contexts [3–5, 10, 12, 14–16]. Consistently, correlation analyses demonstrated strong associations between AMR lesions, notably g and ptc, and dd-cfDNA, as demonstrated previously [2, 12].

In contrast, dd-cfDNA levels were lower and more heterogenous in TCMR biopsies, a pattern that has been reported previously across multiple studies [2, 3, 5, 10–12, 14]. For TCMR-associated lesions, total cfDNA showed a strong influence on dd-cfDNA measurements (Figure 1). As total cfDNA constitutes the denominator of the percentage-based metric, this provides a plausible explanation for the limited increase in relative dd-cfDNA often observed in TCMR. In contrast, absolute dd-cfDNA expressed as copies per milliliter is not affected by recipient-derived cfDNA and therefore showed a stronger association with TCMR-related injury (Figure 2). These observations are strikingly consistent with the limited association between the TCMR/TI index and dd-cfDNA when expressed as a percentage (Figure 2). A potential biological explanation is that infiltrating recipient immune cells undergo cell death within the graft during TCMR, contributing substantially to circulating total cfDNA levels and thereby diluting the relative dd-cfDNA fraction when expressed as a percentage. Independently of this effect on total cfDNA, variability in dd-cfDNA measures among TCMR biopsies may also reflect biological differences in injury mechanisms, whereby some cases exhibit interstitial inflammation accompanied by subtle microvascular injury, leading to increased dd-cfDNA, whereas others are dominated by tubular injury that may preferentially drain into the urine and therefore not be reflected in circulating cfDNA. Notably, in our cohort, seven TCMR cases were grade Ia, six were grade Ib, and none were grade III, resulting in a 25% lower mean of the TCMR/TI index compared to the cohort described by Vaulet et al. [1]. This limited dynamic range likely contributes to the weaker alignment of dd-cfDNA with the TCMR/TI index and to mean CM scores near (and slightly below) zero, rather than clearly positive values. Nonetheless, our findings for the first time provide a plausible mechanistic explanation for why low-grade TCMR is difficult to detect when dd-cfDNA is expressed as a percentage alone.

As expected, patients with Borderline changes had only moderately elevated TCMR/TI and activity indices, which were lower than those with overt TCMR. This is consistent with previous findings indicating that approximately 2/3 of Borderline cases have a molecular phenotype compatible with no rejection [17]. In such ambiguous cases, histological assessment and biomarkers may complement one another, with dd-cfDNA providing additional support in distinguishing true rejection with ongoing graft injury from innocuous infiltrates, indicating which patients might profit from intensified surveillance [11]. Whether dd-cfDNA can distinguish Borderline lesions that require treatment from those that are clinically benign, however, remains speculative and will require confirmation in larger cohorts of borderline cases together with molecular phenotyping.

The consistently low dd-cfDNA levels observed in chronic and non-immune-mediated injury support the concept that dd-cfDNA primarily reflects acute inflammatory injury rather than chronic structural damage. Among chronic Banff lesions, only transplant glomerulopathy (cg) and, to a lesser extent, vascular fibrous intimal thickening (cv), both reflecting chronic vascular and endothelial injury, were associated with increased dd-cfDNA levels, whereas interstitial fibrosis (ci) and tubular atrophy (ct), representing largely scarring, showed no positive association or an inverse relationship. Taken together, chronic graft injury represents a relatively weak driver of dd-cfDNA release, consistent with the findings observed for the chronicity index (Figure 2).

Thus, our dd-cfDNA data, and in particular the novel combined CM score (integrating the percentage and absolute dd-cfDNA) in relation to the rejection-continuum indices help integrate tissue-based and blood-based measures: whereas the continuous histology-based indices quantify the tissue phenotype of inflammation and injury, dd-cfDNA captures the real-time acuity of graft damage in the blood and may further support longitudinal monitoring of clinical response, as indicated previously [11, 18]. Despite the limited dataset, the overall patterns seem consistent with the biological behavior of dd-cfDNA, which reflects acute inflammation that causes cellular injury and death with subsequent release of dd-cfDNA but remains low in chronic structural damage. This supports the concept of a continuum of rejection activity, with microvascular inflammation representing the high-acuity end of the spectrum.

The results presented herein add to the scientific understanding of dd-cfDNA in relation to the specific lesions described by light microscopy and complement the biopsy-derived framework introduced by the publication of Vaulet et al. [1]. Particularly the increase of total cfDNA in TCMR is a novel observation, which offers an explanation, why low grade TCMR is notoriously hard to detect with dd-cfDNA when expressed as percentage only.

Our study has several limitations: (i) the study population is restricted to indication biopsies and does not include protocol biopsies, which may introduce selection bias and limits generalizability to low-pretest-probability surveillance settings and subclinical phenotypes; (ii) both cohorts are European, which constrains external validity across different populations and clinical practices; (iii) TCMR cases were predominantly low grade, which limits inference regarding cfDNA-index relationships in advanced/high-grade (“full-blown”) TCMR; (iv) the combined CM score, while applied here as a pre-specified composite integrating dd-cfDNA percentage and copies/mL, still lacks independent external validation in prospective clinical cohorts; and (v) because our endpoints were the newly proposed continuous rejection indices and Banff ordinal lesion grades, the reported Spearman and R^2^ metrics should be interpreted as descriptive measures of association and not as stand-alone diagnostic performance assessments.

Taken together, these invasive and non-invasive measures may contribute to a unified framework in which histology and circulating biomarkers (dd-cfDNA) converge to quantify active injury, track its trajectory, and potentially aid clinicians to more effectively treat graft inflammation with active injury patterns.

## Data Availability

The raw data supporting the conclusions of this article will be made available by the authors, without undue reservation.
