# Supplementary material for: Donor-Derived Cell-Free DNA as a Non-Invasive Readout of Activity Across the Rejection Continuum
Source: Transpl Int. 2026 Feb 24;39:16099. doi: 10.3389/ti.2026.16099 (PMC12973185; doi:10.3389/ti.2026.16099)
Supplement: Supplementary file 1 [file Supplementaryfile1.docx]

**Supplementary Materials**


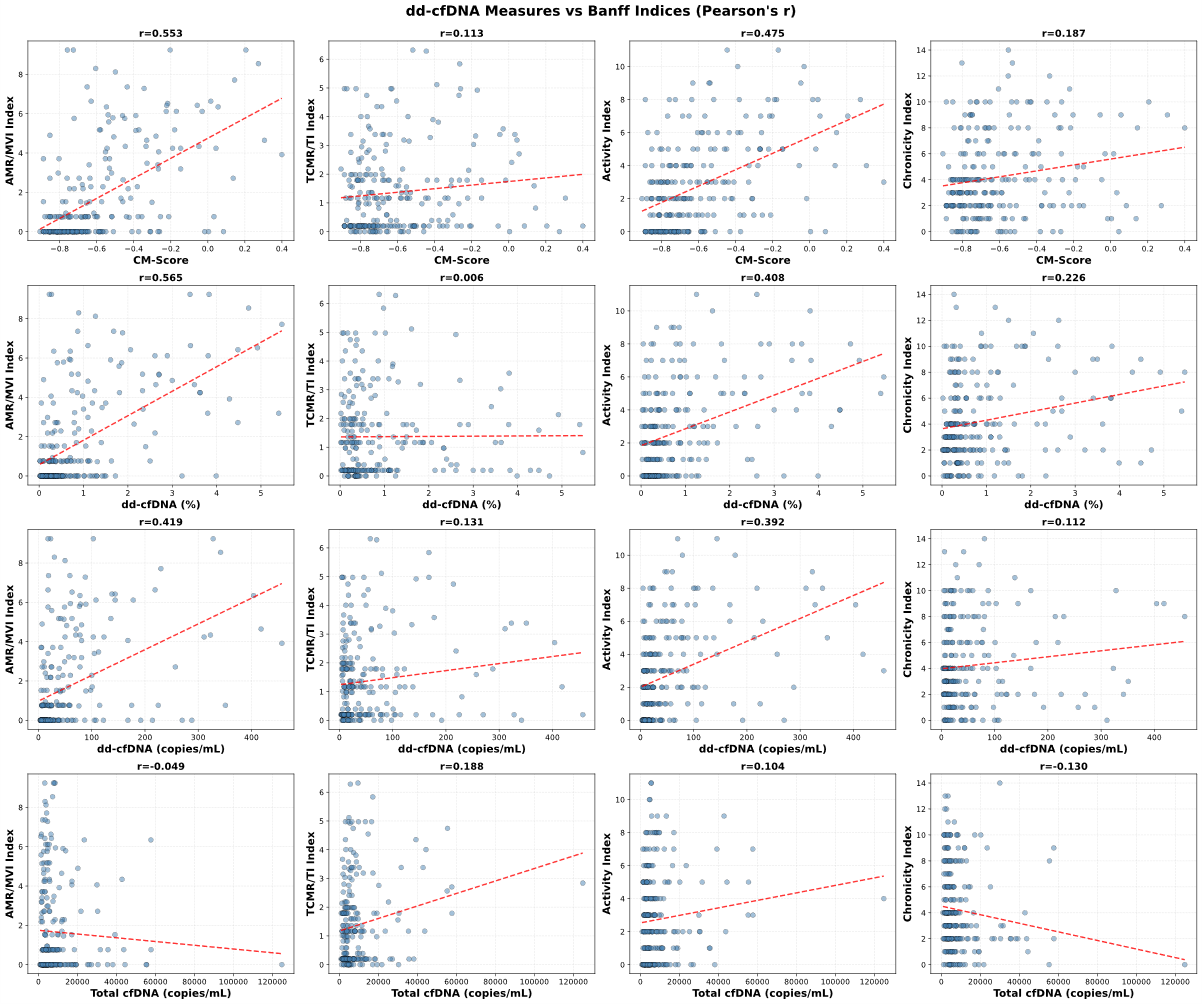


**Supplementary Figure S1 Associations of (donor-derived) cell-free DNA measures with histopathology-derived continuous rejection indices**

Scatterplots showing the relationship between four cfDNA measures, the combined continuous model (CM) score, donor-derived cell-free DNA (dd-cfDNA) fraction (%), absolute dd-cfDNA (copies/mL), and total cfDNA (copies/mL) with the histopathology-derived continuous indices proposed by Vaulet et al. (AMR/MVI, TCMR/TI, global activity, chronicity) in 249 clinically indicated biopsies. Each dot represents one biopsy. Pearson’s r is shown above each panel. The red dashed line indicates a least-squares linear fit provided for visual guidance.
